# Vinegar in Cases of Narcotic Poisoning

**Published:** 1845-09

**Authors:** 


// THE WESTERN JOURNAL.

New Series.—Vol. IV.—No. III.

LOUISVILLE, SEPTEMBER 1, 1845.

### VINEGAR IN CASES OF NARCOTIC POISONING.

Dr. Clapp finds vinegar an excellent adjuvant to emetics, in cases where narcotics have been taken into the stomach in doses to overcome the excitability of that organ. He has succeeded in bringing on vomiting by administering this acid when the emetic was about to fail. He mentioned to us the following instances. A man, in a fit of mental despondency, swallowed an ounce of laudanum on an empty stomach. In about an hour he was visited by Dr. Clapp, and was found insensible, with stertorous, convulsive breathing. Sulphate of zinc was administered to the extent of a hundred grains, and his fauces were tickled with a feather, but vomiting was not induced. The Doctor gave him a pint of vinegar; emesis soon took place, with the relief of all the alarming symptoms.

Two children swallowed a number of seeds of the stramonium at different times. In the case of the first, the ordinary means of exciting emesis were tried ineffectually, and the child died. In the second, vinegar was given, free emesis was the result, and the patient recovered.

These facts are valuable, and a knowledge of them may save the lives of many individuals. We know how often children are sacrificed by the indiscreet use of opiates, and how frequent cases of poisoning by opium, the Jamestown weed, &c., are becoming in this country. If vinegar gives activity to emetics in such cases it is an important auxiliary. Let it be tried.

